# Investigation of the Machinability of the Inconel 718 Superalloy during the Electrical Discharge Drilling Process

**DOI:** 10.3390/ma13153392

**Published:** 2020-07-31

**Authors:** Magdalena Machno

**Affiliations:** Institute of Rail Vehicles, Faculty of Mechanical, Cracow University of Technology, 31–155 Cracow, Poland; magdalena.machno@pk.edu.pl; Tel.: +48-12-374-36-56

**Keywords:** difficult-to-machine material, Inconel 718, electrical discharge drilling, micro-hole

## Abstract

The properties of the Inconel 718 superalloy are used in the manufacturing of aircraft components; its properties, including high hardness and toughness, cause machining difficulties when using the conventional method. To circumvent this, non-conventional techniques are used, among which electrical discharge machining (EDM) is a good alternative. However, the nature of removing material using the EDM process causes the thermophysical properties of Inconel 718 to hinder its machinability; thus, a more extensive analysis of the influence of these properties on the EDM process, and a machinability analysis of this material in a wider range, using more process parameters, are required. In this study, we investigated the drilling of micro-holes into the Inconel 718 superalloy using the EDM process. An experiment was conducted to evaluate the impact of five process parameters with a wide range of values (open voltage, time of the impulse, current amplitude, the inlet dielectric fluid pressure, and tube electrode rotation) on the process’s performance (drilling speed, linear tool wear, the side gap thickness, and the aspect ratio of holes). The analysis shows that the thermal conductivity of this superalloy significantly influences the effective drilling of holes. The combination of a higher current amplitude (*I* ≥ 3.99 A) with an extended pulse time (*t_on_* ≥ 550 µs) can provide a satisfactory hole accuracy (side gap thickness ≤ 100 µm), homogeneity of the hole entrance edge without re-solidified material, and a depth-to-diameter ratio of about 19. Obtaining a high dimensional shape accuracy of holes has an enormous effect on their usability in the structure of the components in the aviation industry.

## 1. Introduction

The Inconel 718 superalloy is one of the most important alloys belonging to the nickel-based superalloy family. This superalloy is mainly used in the aerospace industry to manufacture aerospace parts and gas engine components. In gas engines, about 50% of components are manufactured from nickel-based superalloys. The material is intended for heat treatment recipients, e.g., gas turbine blades, turbine vanes, etc. [1,2,3,4]. The application of Inconel 718 in the aviation industry results from its unique properties, such as high oxidation resistance, corrosion resistance in aggressive environments, resistance to creep, thermal fatigue, very good mechanical properties at both high temperatures (up to 923 °K) and cryogenic temperatures, and its high mechanical strength under these conditions [1,5,6,7,8].

Components of modern gas turbine engines, such as turbine blades, work in high-temperature conditions (in the range of 823.15–1373.15 °K). This causes the production of elements from advanced engineering materials including nickel-based superalloys. Among these superalloys, Inconel 718 is one of the most widely used. To increase the durability of this superalloy, a considerable number of holes are made in the structure of turbine blades (20,000–40,000) with a diameter of 0.3–5 mm and an aspect ratio of (40–600):1 (depth-to-diameter ratio) [9,10,11,12]. These holes are determined as “cooling holes” due to the ability of cooling agents to flow through them (gas or liquid), which reduces the temperature of the component material [13,14]. The dimensional shape accuracy and the quality of the holes’ inner surface affect the efficiency of the cooling process. Non-conventional methods are often chosen to drill the cooling holes, such as the electrical discharge process (EDM) [15,16,17,18].

The combination of the chemical composition of Inconel 718 ensures its excellent mechanical properties. The alloy elements such as Ni and Cr provide resistance to corrosion, oxidation, carburizing, and other damage mechanisms acting at high temperatures. Ni and Cr crystallize as a **γ** phase (face center cubic). Additionally, Al, Ti, Nb, Co, Cu, and W are added to increase mechanical and corrosion resistance. Nb is added to form hardening precipitates *γ*” (Ni_3_Nb, body-centered tetragonal metastable phase). Ti and Al are added to precipitate the intermetallic *γ*’ form (Ni_3_ Ti, Al, simple cubic crystal). These alloy elements characterize a lower influence than *γ*” particles. The two phases (*γ*’ and *γ*”) provide the high strength of the Inconel 718 superalloy (ultimate tensile strength of 1.1 GPa). Further, C is added to precipitate forming MC carbides (M = Ti or Nb). The C content must be low enough to enable Nb and Ti precipitation in the form of *γ*’ and *γ*” particles [3,8,19]. 

The properties of Inconel 718 (such as high hardness, high toughness, very poor thermal conductivity, work hardening, and the presence of highly abrasive carbide particles) make it difficult to machine in the process of constructing aircraft components using conventional methods. The strength of this material at elevated temperatures is quite high, which affects the machining of the superalloy, requiring extreme cutting force. In effect, an enormous amount of heat is delivered to the tool tip. During the machining process, Inconel 718 has a strong tendency to weld to the tool and to form build-up on the edge of the tool. As a consequence, the tool faster wears and the workpiece plastically deforms. Due to these issues, Inconel 718 belongs to the “difficult-to-cut” materials [1,17,20,21,22]. To improve the machinability of the Inconel 718 superalloy by using conventional machining, one should optimize the process parameters using the response surface methodology (RSM) or an analysis-of-variance (ANOVA) [23]. For the above reasons, to machine the Inconel 718 superalloy, non-conventional methods are preferable than conventional machining. Nowadays, electrical discharge machining is among the most effective methods to machine the material [20,24].

When electrodischarge machining the Inconel 718 superalloy, the mechanical properties of the material do not significantly affect the process, which means the forces occurring between the tool and the workpiece surface are negligible or do not take place. The EDM process involves electrical discharges, during which a plasma channel is formed, characterized by a high temperature (about 10,000 °K and locally more than 20,000 °K even for a short pulse duration [25]). In effect, the material is removed by melting, evaporation, and disruption under thermal stresses, which is not affected by its toughness. The heat flux on the workpiece is generally higher than on the tool electrode; therefore, the material removal rate is maximized, and wear of the electrode is minimized. The creation and movements of ions and electrons inside the plasma channel allow electricity to pass through the electrode and the workpiece. The bombardment of ions is related to the highly concentrated electricity flow and conduction of heat from the plasma to the electrodes, which heats the material on both the workpiece and tool electrode sides. During the discharge time, a bubble of vapor is created around the plasma due to the high temperature. The plasma enlarges during the discharge. When the discharge ends, the plasma collapses, and the material is then ejected. The rapid cooling during the pulse-off time leads to the formation of spherical debris, a typical shape that is obtained when a liquid is rapidly solidified [26,27,28].

The phenomena that occur during the removing process of material using the EDM process cause that the properties of the workpiece material such as thermal conductivity, density, melting point, evaporation temperature, and coefficient of thermal expansion significantly effect this process. The enthalpy of vaporization and boiling point of the tool electrode material influence the evaporating intensity of the workpiece material. The increase in these properties decreases the volume of the evaporated material of the workpiece. The tool material is affected similarly during single discharge; however, the occurrence of thermal expansion causes the stresses in the horizontal plane. This is a result of the inability of the material to expand in this plane due to the presence of a material layer with a lower temperature, which does not allow the deformation [28].

The consequence of spark erosion is a heterogeneous spreading of the heat onto the surface workpiece and the working electrode under the pressure of the plasma channel formed during electrical discharge; therefore, the main factor influencing the erosion process is the thermal conductivity of the electrodes’ material (workpiece and tool) [28]. In the case of Inconel 718, its thermal conductivity is low (8.9 W/(m·°K) at 298 °K [8]), which significantly affects the process of material removal. This causes a lower amount of heat to be delivered to the workpiece material. Consequently, a considerable amount of heat penetrates the tool electrode material and occurs in the gap area (which results in insufficient flushing of the interelectrode gap). The difficulties of the flushing efficiency take place especially during the electrical discharge drilling of deep holes with a diameter of less than 1 mm [29]. This leads to excessive tool wear and process instability. Moreover, the presence of high-temperature conditions and erosion products (eroded particles and bubbles) changes the conditions occurring in the gap area. These conditions can contribute to secondary/abnormal discharges (such as arcing and short circuits), decreasing the dimensional shape accuracy of the hole. Additionally, in [30], it was shown that the spherical eroded particles can join into the debris chains, which are more difficult to remove from a narrow gap area. 

In the case of the EDM drilling of high-aspect ratio holes (above 20:1) with a diameter of less than 1 mm, effective gap flushing is a challenge [31]—even during the application of a high volumetric flow rate (25 L/h) [32]. To prevent secondary discharges between debris and the hole sidewall, a satisfactory approach is to cover the sidewall of the tool electrode with a non-conductive coating. The best results are obtained for Perylene C-coated tools. This approach enables us to reach an aspect ratio of 126:1 within 1 h for micro-holes with a diameter of 0.18 mm and a depth of 10 mm. Additionally, the tool wear is two times lower when using an insulated electrode compared to using an uncoated electrode [33]. To minimize the accumulation of debris at the hole bottom, there are several flushing methods, such as internal, external, suction-assisted flushing, flushing with different electrode movements, or vibration-supported flushing [33,34].

In [35], the authors investigated the influence of three different electrode materials, such as brass, copper, and copper tungsten (CuW), on Inconel 718 machinability with the use of hybrid electrical discharge and arc machining (HEDAM). The analysis shows that for all current settings, the brass electrode allows one to achieve the highest material removal rate; however, the copper tungsten electrode provides the lowest electrode wear. The experimental research also included an investigation of the average surface roughness (*Ra*) and surface characteristics of the electrodes after machining. The authors mentioned in the impact analysis of the thermal properties of Inconel 718 and the tool electrode materials on the process’s performance that brass and copper allow one to obtain less *Ra* compared to using the CuW electrode. 

The above analysis shows that the machinability of the nickel-based superalloys demonstrates the difficulty of machining by conventional methods; however, the thermophysical properties of these superalloys also contribute to the difficulties of hole drilling with the use of the EDM process as well (especially the drilling of deep holes with a small diameter—less than 1 mm). The EDM process is a non-contact method and is preferable for machine materials determined as “difficult–to–cut”; however, the presence of high temperatures (the heating generated from electrical discharges) during the material removal hinders the process. The main factor causing the decreased machinability of Inconel 718 is its low thermal conductivity [35]. The differing behavior of the superalloy with increasing temperature has a significant influence on the effectivity of material removal; however, this behavior is required to achieve good hole accuracy and high EDM machining performance. Obtaining high dimensional shape accuracy of holes is a crucial issue for their application. The majority of papers concerning machining holes in a smaller range focus on the impact of the material’s thermophysical properties on the allowance removal process. The thermophysical properties of Inconel 718 in a wider range can significantly influence the appropriate selection of the machining parameters and improve the machinability of the Inconel superalloy with the use of EDM. Due to the specific behavior of this superalloy with increasing temperature, the research should be directed toward the analysis of a greater number of process parameters, including a wider range of values.

In this paper, we present the analysis of the results of the experiments on electrical discharge drilling of the Inconel 718 superalloy. In this study, we aimed to: investigate the machinability of Inconel 718 by using the EDM process to enable the drilling of holes with a high aspect ratio and satisfactory dimensional shape accuracy; to determine the appropriate influence of process parameters on the process’s performance; and to investigate a wider range EDM on Inconel 718—the experimental research includes the influence analysis of five machining parameters on the process’s performance. The analyzed process parameters involve parameters like open voltage, time of the impulse, current amplitude, the inlet dielectric fluid pressure, and tube electrode rotation; however, the process’s performance was analyzed in terms of drilling speed, linear tool wear, the side gap thickness, and the aspect ratio of holes.

## 2. Materials and Methods 

### 2.1. Workpiece and Tool Electrode Material

**Figure 1:**
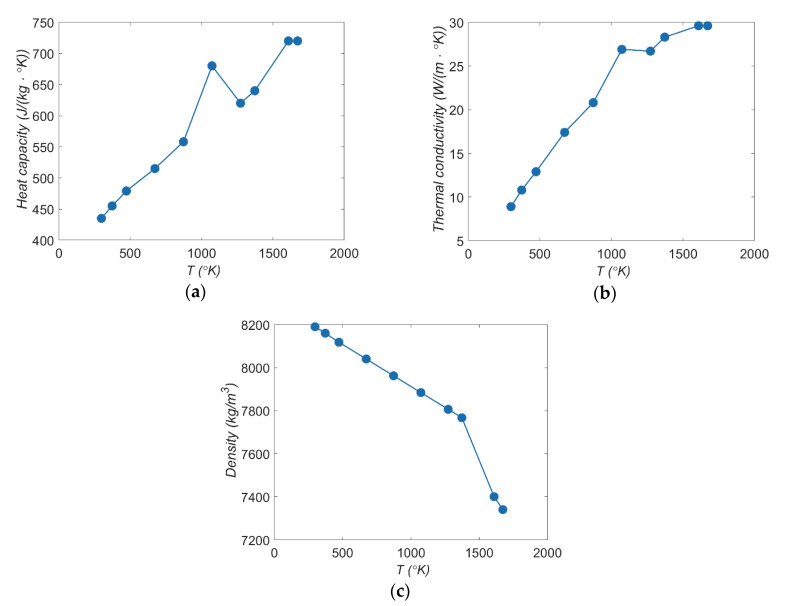
Thermophysical properties of Inconel 718 superalloy according to the increase in temperature *T*: (**a**) heat capacity; (**b**) thermal conductivity; (**c**) density [8].

The nickel-based superalloy Inconel 718 was selected as a workpiece material with the dimensions 30 × 25 × 10 mm. The chemical composition and the main mechanical properties of this material are presented in Table 1 and Figure 1a–c.

The properties of the electrodes’ material (tool electrode and workpiece) significantly affect the electro-erosion process. The most influencing properties of the electrodes’ material are thermal conductivity, melting point, evaporating point, and the heat-expansion coefficient; however, the main factor affecting the electrodischarge process is the thermal conductivity of the electrodes’ material. In the case of metal alloys, the values of thermal conductivity are usually less than metal elements, including its chemical composition (e.g., the thermal conductivity of nickel is 92.4 W/(m∙°K) at 293.15 °K). In addition, the thermal conductivity of the metal alloy behaves differently with increasing temperature in comparison to including its chemical composition. For the Inconel 718 superalloy, the thermal conductivity at 298.15 °K amounts to 8.9 W/(m∙°K) and increases with the increase in temperature (Figure 1b); however, the thermal conductivity of pure nickel decreases with increasing temperature.

**Figure 2:**
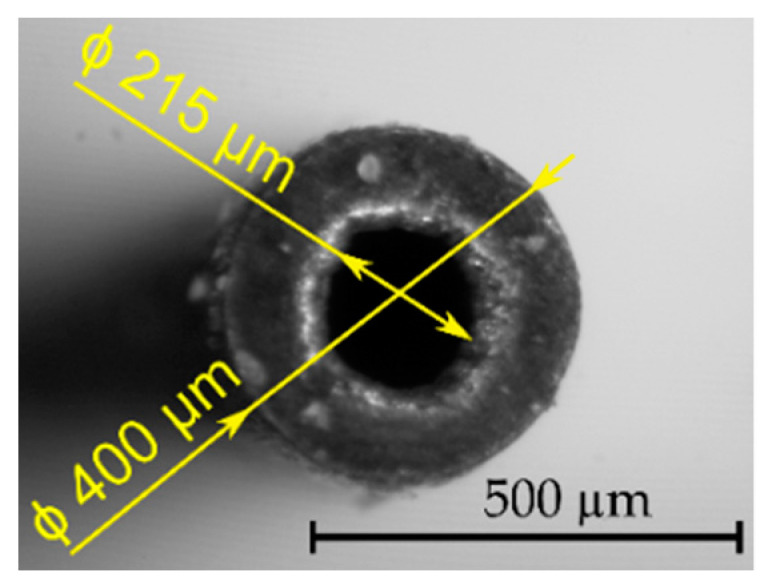
The tool electrode tip.

In the experimental research of electrical discharge drilling (EDD) a tool electrode was used as a single-channel tube electrode made of copper (Figure 2). The material of the tool electrode is classified as one with the most electrical conductivity (up to 60.9 MS/m). The selected thermophysical properties for copper are presented in Table 2.

According to [37], a copper tube electrode is the best choice for the electric discharge deep hole drilling of Inconel 718, which allows one to obtain a high material removal rate and low surface roughness. Additionally, the single-channel electrode provides comparatively better removal rates and a lower electrode wear ratio than multi-channel electrodes [38]. These determine the selection of this kind of tool electrode. 

### 2.2. Experiment Design

**Figure 3:**
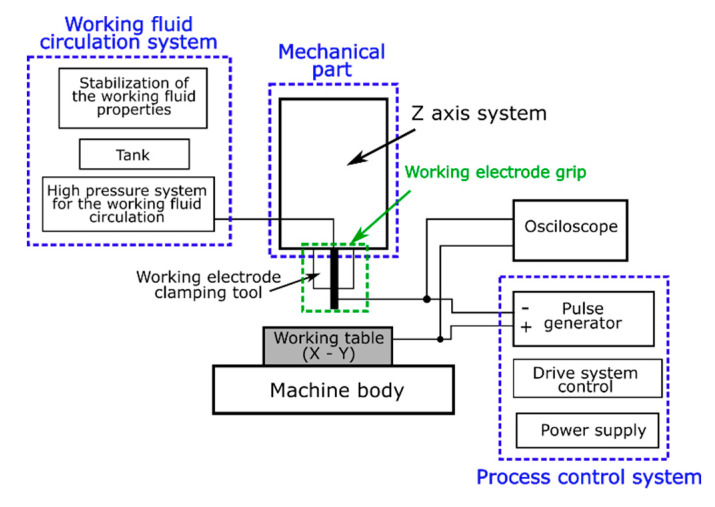
Scheme of the test stand and its main functional units.

The electrical discharge drilling was carried out on the experimental test stand shown in Figure 3. The purpose of the tests was to examine the impact of machining parameters (open voltage, pulse time, current amplitude, inlet dielectric fluid pressure, and tube electrode rotation) on the process’s performance (drilling speed and linear tool wear) and the dimensional accuracy of the holes (aspect ratio hole and the side gap thickness). Table 3 presents the data on the drilling process, and Table 4 presents the adopted ranges of values. The experiments were performed according to the theory of the experiment using a five-level rotatable research plan that included 32 experimental tests with six repetitions in the center of the research plan. The results of the experiments are shown in Table 5. The *Matlab* software (The MathWorks, Inc., R2019a, Natick, MA, USA) was applied to investigate the relationship between process parameters and output parameters.

**Figure 4:**
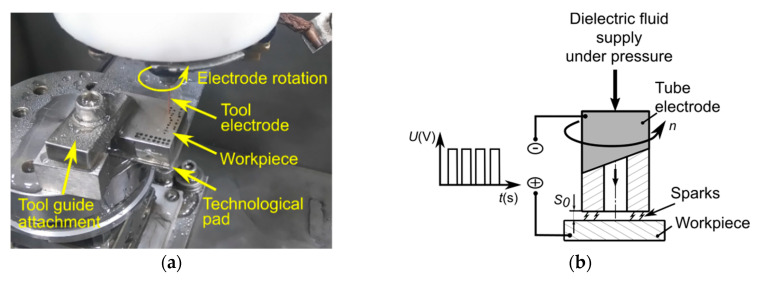
(**a**) A photograph of the experimental setup; (**b**) the scheme of the electrical discharge drilling process.

The following constant parameters were assumed: initial interelectrode gap thickness (*S*_0_ = 50 μm), drilling time of each hole (*t_drilling_* = 45 min or shorter in the case if through hole was obtained faster), pulse off time (*t_off_* = *t_on_*), and deionized water as the dielectric fluid (with the electrical conductivity in the range 1.00–8.5 µS/cm). The experimental setup is presented in Figure 4a. The dielectric fluid was flushed down to the gap zone through the interior hole of the tube (Figure 4b).

The drilling speed *v* is calculated from the following equation [39]:

*v* = *h*/*t*_*drilling*_, (1)

where *h* is the hole depth and *t_drilling_* is the drilling time.

The linear tool wear *LTW* is calculated according to the formula

*LTW* = (*h*_*tool*_/*h*)·100%, (2)

where *h_tool_* is the shortening of the electrode.

The aspect ratio hole *AR* is given by the following Equation [40]:

*AR* = *h*/*D*_*average*_, (3)

where *D_average_* = (*D_top_* + *D_bottom_*)/2 is the average of the hole diameters, *D_top_* is the average top diameter, and *D_bottom_* is the average bottom diameter. In case a hole was not obtained, *D_bottom_* equals the outer diameter of the tool electrode. The values of *D_top_* and *D_bottom_* are the mean values from 10 measurements. Measurements of the diameters were performed with the use of the K-401 stereo microscope with a common main objective (CMO) Infinity optical system (Motic, Richmond, BC, Canada) and equipped with a Moticam 2300 digital camera with the MoticImages Plus system (Motic, Richmond, BC, Canada). 

**Figure 5:**
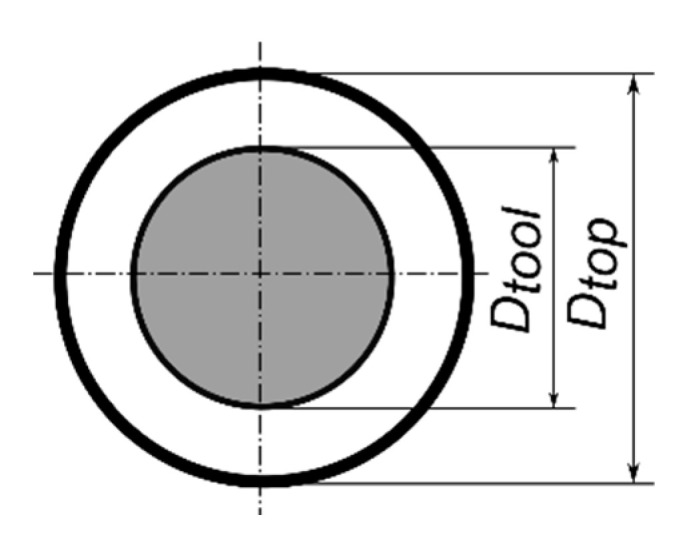
Scheme of determining the side gap thickness.

The side gap thickness *SG* is calculated according to the formula [40] (Figure 5)

*SG* = (*D*_*top*_ − *D*_*tool*_)/2. (4)

where *D_tool_*—outer diameter of the tool electrode.

The standard deviation values of the result values for repeating tests (27–32 in the research plan, Table 5) are greater (standard deviation—*std* for: drilling speed, (µm/s)—*v*, *std* = 0.73; linear tool wear—*LTW*, *std* = 1.06; aspect ratio—*AR*, *std* = 1.43; side gap thickness, (µm)—*SG*, *std* = 40.06). The greatest value of *std* is obtained for *SG*. This can be the result of various temperatures of deionized water, which is related to its electrical conductivity. In [31], the analysis of the results shows that the deionized water temperature affects the condition at the machining area, and the change in its electrical conductivity. The application of a higher initial working fluid temperature (about 313.15 °K) improves the dimensional shape accuracy of drilled holes (the side gap thickness decreases by about 40%). This is the result of removing the allowance by EDM, accompanied by reinforced electrochemical dissolution. 

In the experimental research performed in this study, we did not focus on the influence of the deionized water properties on the process. The working temperature was also not studied, i.e., fluid in tank and out-flowing from the tube electrode were not measured; however, the electrical conductivity of the working fluid was measured at the beginning of each research day. Moreover, in-tank there is a high-pressure system, which increases the temperature of the working fluid. This could explain drilling tests 27–32, where variable electrical conductivity of deionized water occurred, contributing to differences in the obtained result values.

## 3. Results

To investigate the impact of the machining parameters on the process’s performance, the *Matlab* software was employed. A second-degree polynomial (with constant, linear, and square terms) was used as the function. After eliminating the non-significant factors in the obtained regression Equations *v* (*U, t_on_*, *I, p, n*), *LTW* (*U, t_on_*, *I, p, n*), *AR* (*U, t_on_*, *I, p, n*), and *SG* (*U, t_on_*, *I, p, n*), these are described as Equations (5)–(8), respectively.

*v* (*U, t_on_*, *I, p, n*) = 14.23 + 0.074 *U* + 0.0072 *t_on_* − 6.40 *I* − 5.08 *p* + 0.014 *n* − 0.0000043 *t_on_*^2^ + 1.40 *I*^2^ + 0.039 *p*^2^, (5)

*LTW* (*U, t_on_*, *I, p, n*) = 20.42 − 1.16 *U* + 0.031 *t_on_* + 2.73 *I* + 9.49 *p* + 0.072 *n* + 0.0066 *U*^2^ − 0.000013 *t_on_*^2^ − 0.7 *p*^2^ − 0.00 13 *n*^2^, (6)

*AR* (*U, t_on_*, *I, p, n*) = − 17.87 + 0.8 *U* − 0.0053 *t_on_* − 1.2 *I* − 3.08 *p* + 0.058 *n* − 0.0043 *U*^2^ + 0.000005 *t_on_*^2^ + 0.42 *I*^2^ + 0.23 *p*^2^ − 0.000087 *n*^2^, (7)

*SG* (*U, t_on_*, *I, p, n*) = − 707.97 + 0.25 *t_on_* + 220.14 *I* + 183.47 *p* − 0.35 *n* − 0.00025 *t_on_*^2^ − 37.68 *I*^2^ − 13 *p*^2^ + 0.00054 *n*^2^. (8)

Analysis of the results shows that the values of the *R*-squared statistic (*R*^2^) for drilling speed *v*, the linear tool wear *LTW*, the aspect ratio hole *AR*, and the side gap thickness *SG* were 0.87, 0.48, 0.53, and 0.31, respectively. Differences between the *R*-squared statistic and the adjusted *R*-squared statistic are smaller than 0.2. The high value of the coefficient *R*^2^ (near value 1) for *v* shows that the data of the regression model are very close to the experimental data. The lower values of the coefficient *R*^2^ for *LTW*, *AR*, and *SG* are the result of analyzing five input parameters on the response and smaller differences between the obtained result values.

**Figure 6:**
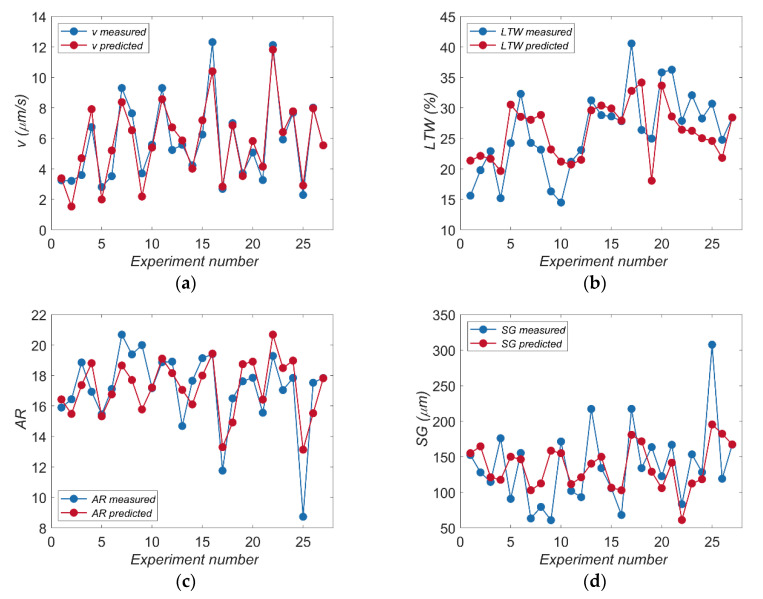
Comparison of measured and predicted values for: (**a**) drilling speed—*v*; (**b**) the linear tool wear—*LTW*; (**c**) the aspect ratio hole—*AR*; (**d**) the side gap thickness—*SG*.

The result of the *F* statistic indicates for the whole of the developed regression models that the *p*-values are less than 0.05 (i.e., 95% of the confidence level), which confirms that the models obtained are statistically significant. Comparisons between the results of experimental studies and the values calculated based on the developed regression models for *v*, *LTW*, *AR,* and *SG* are presented in Figure 6a–d.

### 3.1. Influence of Voltage, the Pulse Time Duration, and Current Amplitude on the EDD Process

**Figure 7:**
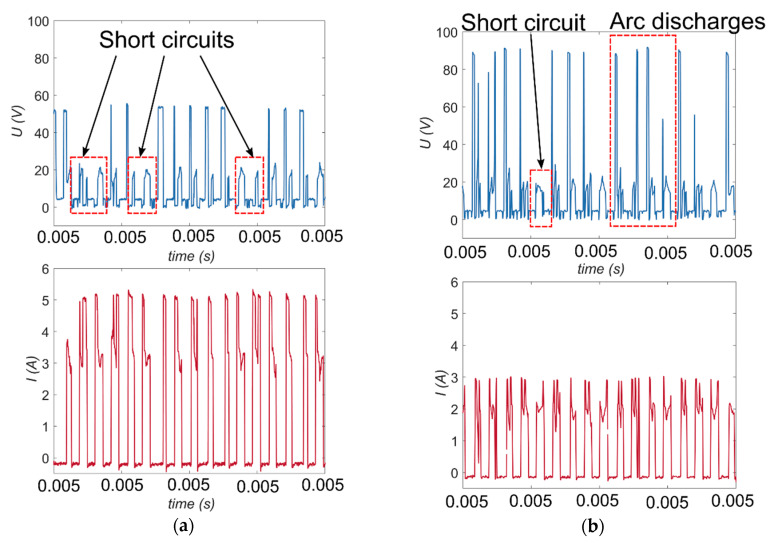
The recorded voltage and current waveforms for the following process parameters: (**a**) *U* = 60 V, *t_on_* = 550 µs, *I* = 3.33 A, *p* = 7 MPa, *n* = 300 rpm; (**b**) *U* = 100 V, *t_on_* = 550 µs, *I* = 2.00 A, *p* = 7 MPa, *n* = 300 rpm.

An analysis of the results shows that the applied lower value of voltage (*U* = 60 V) and a lower current amplitude (*I* = 2.00 A) contribute to obtaining a too high value of the side gap thickness (*SG* = 217 µm and *SG* = 167 µm, respectively) and a decrease in drilling speed (*v* = 2.69 µm/s and *v* = 3.27 µm/s, respectively). The analysis of the registered voltage waveforms *U(t)* for these tests (Figure 7a,b) reveals that the ignition delay time *t_d_* is either too short or equals zero. This indicates that the arc either discharged or short-circuited in the gap area. The result of the phenomenon can be a value that is too small for the interelectrode gap thickness or eroded particles accumulating at the hole bottom. The arc discharges and/or short circuits are undesirable phenomena during the electrical discharge drilling process. The presence of the undesirable phenomena also confirms the current waveforms *I(t)* (Figure 7a), in which the values are higher than the applied values. For the test applying *U* = 60 V, the recorded values of the current amplitude are about 5 A when the applied value of *I* equals 3.33 A.

**Figure 8:**
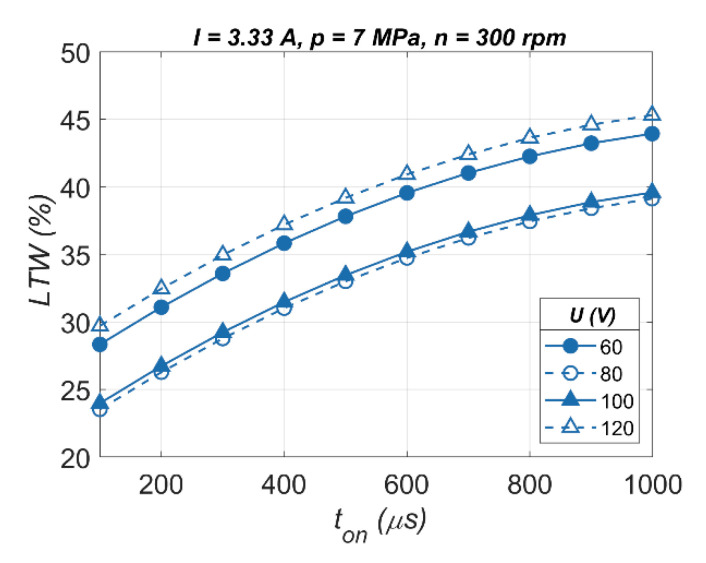
The impact of various values of the open voltage on the linear tool wear *LTW*.

The arc discharges and/or short circuits result in an unstable erosion process, making it difficult to remove material, with excessive tool wear and a decrease in drilling performance. This is noted by the lower obtained values of drilling speed and also the higher values of the linear tool wear *LTW* at about 40%. Figure 8 shows that the *LTW* has similar values for applying *U* = 60 V and *U* = 120 V. For the test using *U* = 120 V, the drilling speed was high, *v* = 7.00 µm/s, confirming that material was removed and tool wear was normal.

In the case of a lower value of *U* and lower values of *I*, the amount of energy of the electrical discharges can be too small to remove sufficient material. The low thermal conductivity of Inconel 718 can prevent the sufficient heating of the machined surface, which causes melting and re-solidifying material. Figure 8 shows the pulse time duration and the increase in *LTW*; a longer impulse time results in a longer electrical discharge time (arc discharges/short circuits for *U* = 60 V) in a single pulse.

**Figure 9:**
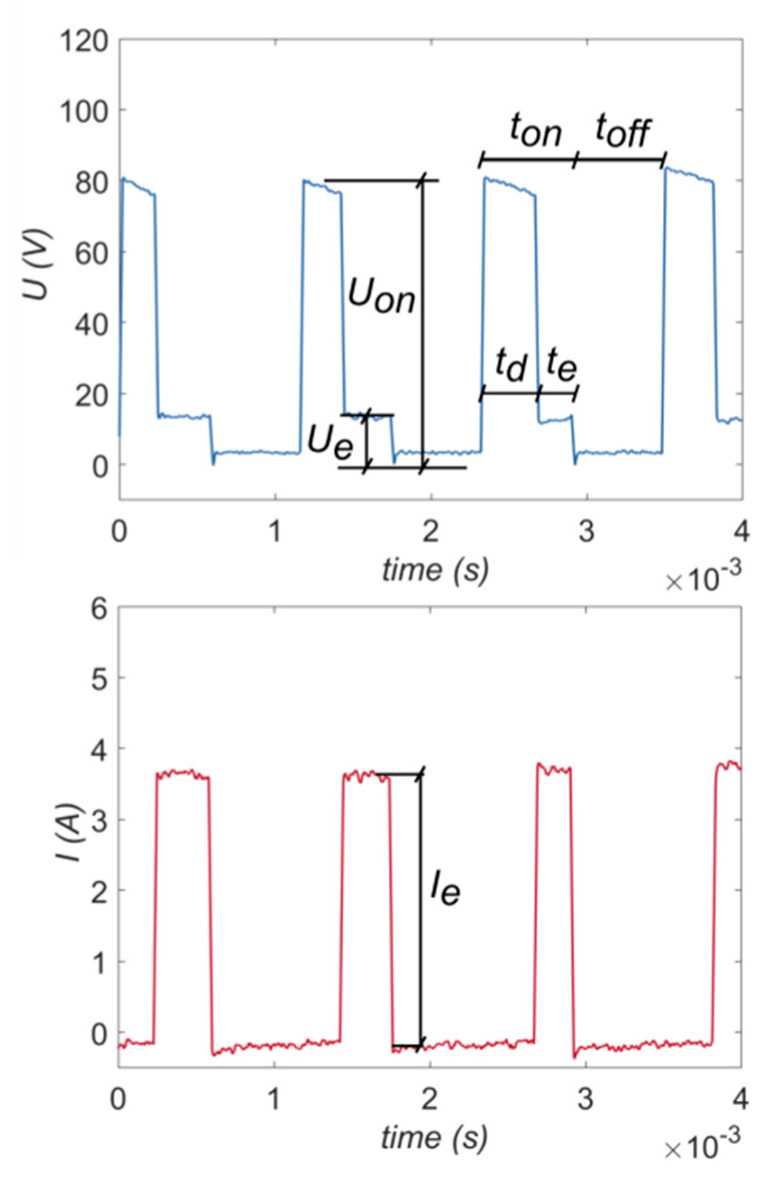
The recorded current and voltage waveforms.

Figure 9 shows typical current and voltage waveforms registered for the investigation of EDM with the use of deionized water as a dielectric fluid. During the EDM process, while the supply voltage *U_on_* drops to the discharge voltage *U_e_*, there is an increase in the discharge current *I_e_* during the pulse time *t_on_*.

Below, the parameters presented in Figure 9 are characterized [41,42,43]:

- *U_on_*—the relevant open circuit voltage, which is the system voltage when the EDM circuit is in the open state, and the energy has been built up for discharge;
- *U_e_*—the discharge voltage (usually 20–30 V);
- *I_e_*—the maximum peak current discharging;
- *t_on_*—the duration of pulse time, the time required for the current to rise and fall during discharging;
- *t_off_*—the duration of the time interval;
- *t_d_*—the ignition delay time;
- *t_e_*—the duration of the appropriate EDD process.

**Figure 10:**
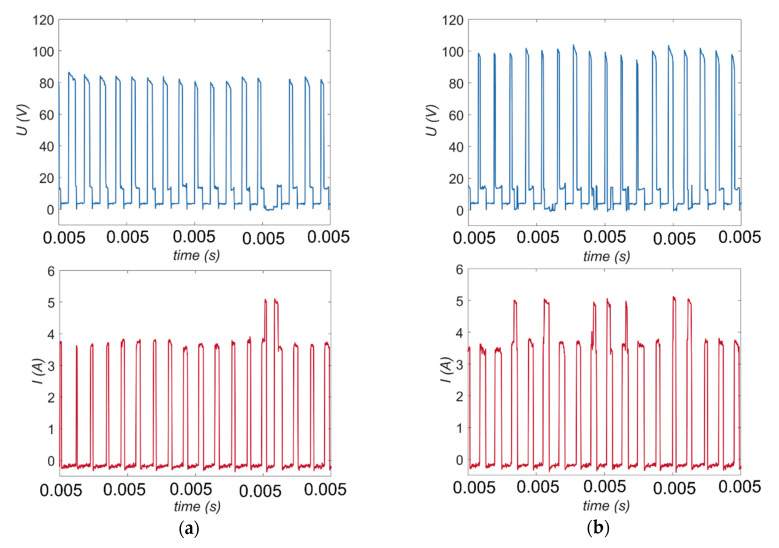
The recorded voltage and current waveforms for the applying open voltage: (**a**) *U* = 100 V; and (**b**) *U* = 120 V; *t_on_* = 550 µs, *I* = 3.33 A, *p* = 7 MPa, *n* = 300 rpm.

For applying higher values of the open voltage (*U* = 100 V and *U* = 120 V), the recorded waveforms of current and voltage are appropriate for the EDM process using deionized water (Figure 10a,b).

**Figure 11:**
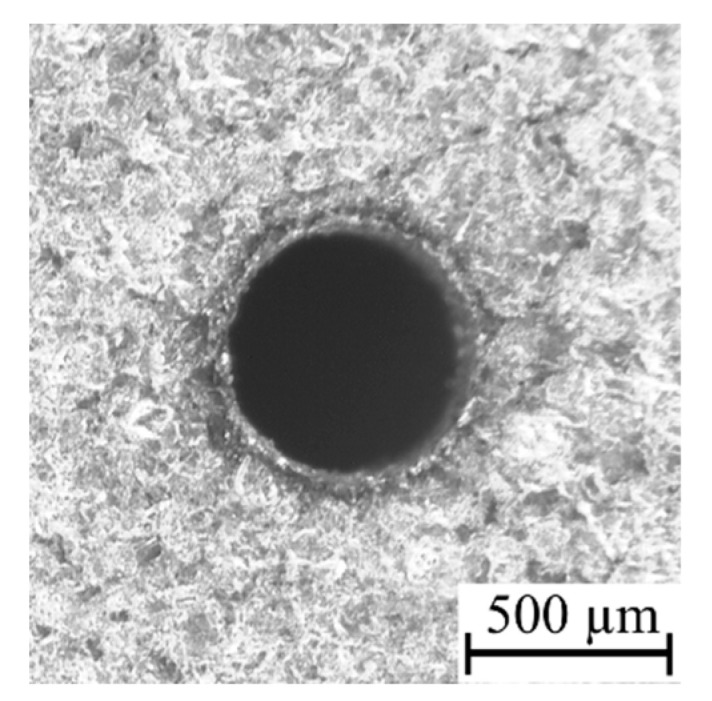
The entrance diameter of a micro-hole fabricated by the open voltage *U* = 100 V and *t_on_* = 550 µs, *I* = 3.33 A, *p* = 7 MPa, *n* = 300 rpm.

**Figure 12:**
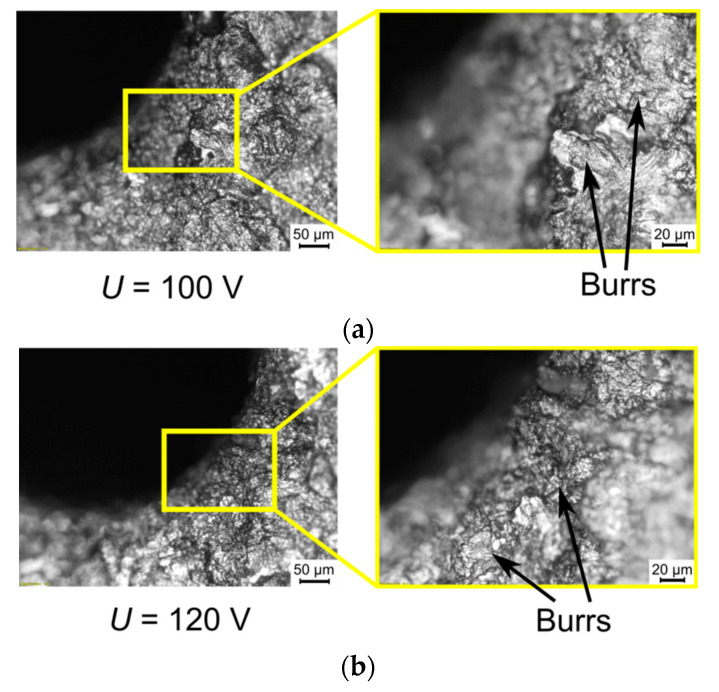
The edge of entrance diameter of micro-hole fabricated by various values of the open voltage: (**a**) *U* = 100 V; and (**b**) *U* = 120 V; the other machining parameters: *t_on_* = 550 µs, *I* = 3.33 A, *p* = 7 MPa, *n* = 300 rpm.

Applying a higher open voltage allows for the faster heating of deionized water during the ignition delay time, which increases the electrical conductivity of the deionized water. For this reason, during *t_d_*, electrochemical dissolution can be enhanced and significantly influence the process. The presence of electrochemical dissolution in the single impulse improves the efficiency of the EDM process, particularly for obtaining higher values of the drilling speed and higher values of the aspect ratio hole (for applied *U* = 100 V: *v* = 5.33 µm/s, *AR* = 17 and for applied *U* = 120 V: *v* = 7.00 µm/s, *AR* = 16). In addition, the dimensional accuracy of the obtained holes is also improved by an excessive electrochemical dissolution. The values of the side gap thickness are 130–160 µm and the edges of the entrance diameter are characterized by a small number of burrs (Figure 11); however, for the test applying *U* = 120 V, the burrs that formed are smaller and smoother in comparison to the test applying *U* = 100 V, which is the result of the reinforced electrochemical reaction improving accuracy (Figure 12a,b).

To confirm the occurrence of electrochemical dissolution in the single impulse, an additional experiment was conducted using the electrochemical machining process (ECM). Table 6 presents the machining conditions.

**Figure 13:**
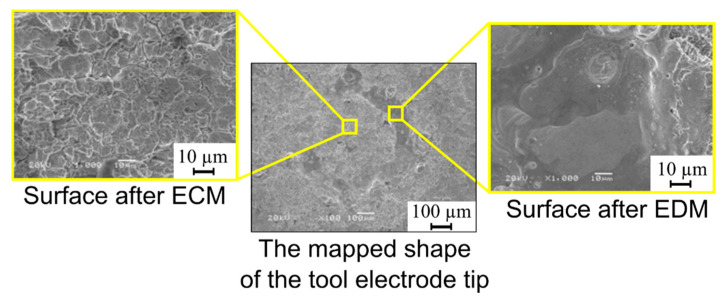
SEM images after the ECM test.

The recorded current and voltage waveforms showed that the current amplitude during the time test increased from 0.04 to 0.4 A. This indicated the increase in electrical conductivity of the deionized water by heating. At the end of the test, the temperature of the deionized water was so high that it caused conditions to occur such that electrical discharge resulted in the interelectrode gap area. Figure 13 shows images taken via an electron microscope (SEM) (manufactured by JEOL Ltd. (Tokyo, Japan)). Figure 13 shows a trace mapping of the tool electrode tip and the surfaces after the ECM and EDM processes.

The duration of the pulse time and the current amplitude influence the amount of thermal energy delivered to the workpiece, which contributes to the melting and evaporation of material. During electrical discharge, part of the material should evaporate, part of the material should be melted, and part of the material should be removed due to thermal stresses. In this case, the Inconel 718 superalloy was used as the workpiece, which is characterized by a low thermal conductivity, and as such, the thermal energy delivered to the workpiece should be high enough. In addition, the thermal conductivity of Inconel 718 increases with increasing temperature (Figure 1b). To increase the speed of material removal requires an appropriately long pulse time. 

**Figure 14:**
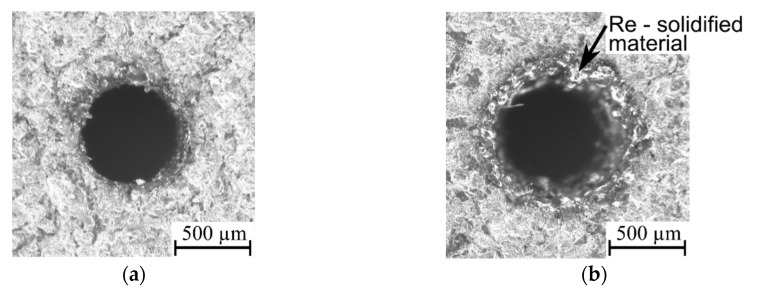
The entrance diameter of a micro-hole fabricated by the pulse time duration: (**a**) *t_on_* = 325 µs and *I* = 2.66 A, *U* = 100 V, *p* = 6 MPa, *n* = 400 rpm; (**b**) *t_on_* = 775 µs and *I* = 2.66 A, *U* = 100 V, *p* = 6 MPa, *n* = 400 rpm.

For the lower value of the current amplitude (*I* = 2.66 A), the amount of energy is too small to remove material appropriately (Figure 14a). This causes the occurrence of burrs and an increasing side gap thickness at the edge of the entrance diameter (*SG* = 171 µm). Additionally, an extension of the pulse time contributes to heating a larger amount of material, but a lower current amplitude does not allow the removal of all the material. In effect, a large amount of material is only melted and re-solidified (Figure 14b), which decreases the dimensional accuracy of the hole (*SG* = 217 µm). Moreover, during the drilling test, the recorded voltage–current waveforms indicate the presence of short circuits, which confirms the worsening of the process’s stability.

**Figure 15:**
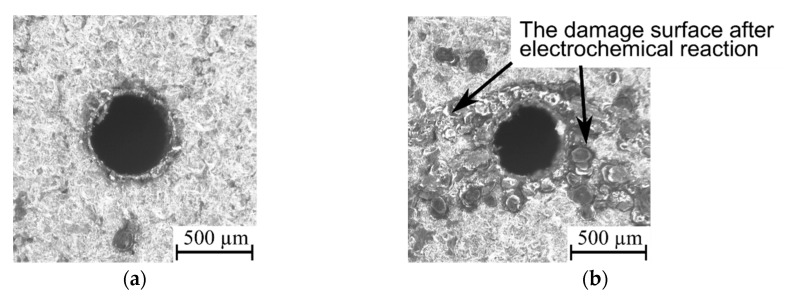
The entrance diameter of a micro-hole fabricated by the pulse time duration: (**a**) *t_on_* = 325 µs and *I* = 3.99 A, *U* = 100 V, *p* = 8 MPa, *n* = 400 rpm; (**b**) *t_on_* = 775 µs and *I* = 3.99 A, *U* = 100 V, *p* = 8 MPa, *n* = 400 rpm.

The higher value of current amplitude (*I* = 3.99 A) delivers enough energy to the workpiece, which results in obtaining a lower value of side gap thickness (*SG* = 102 µm); the accuracy of the entrance diameter is also improved (Figure 15a). An additional extension of the pulse time, resulting in higher thermal energy being delivered to the workpiece, reduces the value of *SG,* which equaled 68 µm (Figure 15b). The above results show that the thermal energy is sufficient to remove appropriate allowance and to obtain a high accuracy of the hole without burrs and re-solidified material. The control of the heating conditions during the EDM process is difficult, especially for drilling aspect ratio holes. When drilling a long hole with a diameter of less than 1 mm, the effective flushing of the gap area is difficult to reach. This causes the heating conditions in the gap area to be higher. At the bottom of the hole, the accumulation of erosion products (debris and bubbles) changes the conditions in the machining gap. Accumulated debris and bubbles contribute to the secondary/abnormal discharges, extending the exit diameter.

The higher current amplitude also caused faster heating of the deionized water in the gap area and significantly increased its electrical conductivity. The higher electrical conductivity of the deionized water reinforced the electrochemical dissolution during the EDM process. Serious damage on the surface around the top diameter is seen in Figure 15b; however, an excessive electrochemical dissolution increases the process efficiency. The obtained drilling speed for this test is the highest value (*v* = 12.32 µm/s) in comparison to other tests; the aspect ratio hole is also high for this drilling test (*AR* = 19).

**Figure 16:**
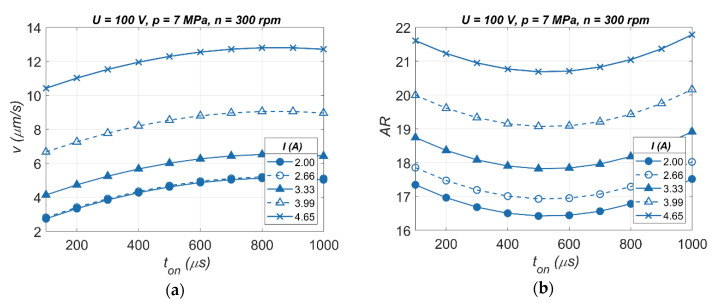
The impact of various values of the current amplitude on: (**a**) the drilling speed *v*; (**b**) the hole’s aspect ratio *AR*.

From the analysis of the relationship of the drilling speed and the hole’s aspect ratio, for various values of *I* (Figure 16a,b), applying the current amplitude above 3.99 A and extending the pulse time allows for obtaining higher values of *v* and *AR*.

### 3.2. Influence of the Inlet Dielectric Fluid Pressure and the Tool Electrode Rotation on the EDD Process

In the EDD process, one crucial issue is the flushing of the gap area and the evacuation of the erosion products to provide process stability. The accumulation of debris and bubbles at the hole bottom decreases the process performance and worsens the hole accuracy. An accumulation of eroded particles at the hole bottom can create a debris chain [26]. Debris chains are more difficult to remove from the gap area. Additionally, the joined debris reduces the machining area, which leads to secondary discharges in a wider range. The reduction in the gap area also causes the possibility of short circuits and arc discharges. To improve the flushing efficiency, we used higher inlet dielectric fluid pressure and higher tool electrode rotation. The results analysis in this study also shows good results for higher values of these process parameters on the drilling efficiency.

**Figure 17:**
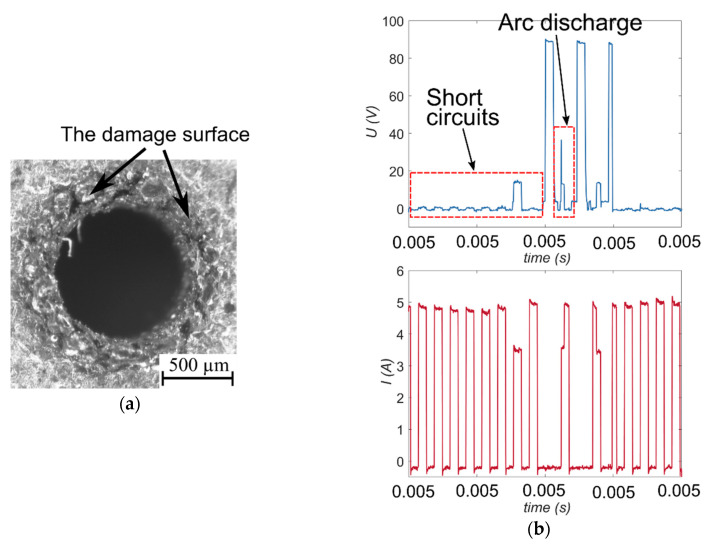
(**a**) The entrance diameter of a micro-hole fabricated with the application of the lower tool electrode rotation *n* = 100 rpm and *U* = 100 V, *t_on_* = 550 µs, *I* = 3.33 A, *p* = 7 MPa; (**b**) the recorded voltage and current waveforms for this test.

The tool electrode rotation also influences the accuracy of the entrance diameter. The applied lower value of the tool electrode rotation (*n* = 100 rpm) significantly decreases the dimensional shape accuracy of the hole (*SG* = 308 µm) and homogeneity of the top diameter edge (Figure 17a). This shows that the difficulties removing eroded particles were already present at the beginning of the process. Inefficient flushing of the machining area contributes to reducing the area of a narrow interelectrode gap. The analysis of the recorded waveforms of current and voltage indicates the presence of short circuits and arc discharges during drilling (Figure 17b). For the test, the drilling speed is low (*v* = 2.29 µm/s) and the hole aspect ratio was the lowest value obtained from all experiments (*AR* = 9); however, the higher tool electrode rotation (*n* = 500 rpm) improved the accuracy of the top diameter (*SG* is decreased about 60%) and increased *AR* 4-fold.

## 4. Discussion

The analysis shows that the electrical discharge drilling of micro-holes with a diameter of less than 1 mm in the Inconel 718 superalloy is difficult when trying to obtain satisfactory dimensional and shape accuracy of the drilled holes and high process efficiency. Electrical discharge machining is dedicated to the machining of “difficult-to-cut” materials, which involve the chromo-nickel alloy (including Inconel 718); however, the superalloy’s properties, such as the thermal conductivity or the heat capacity, when temperatures increase during the machining process, contribute to difficulties during machining with the use of EDM. Additionally, for the electrical discharge drilling process of high-aspect ratio micro-holes, debris accumulates at the hole bottom, causing difficulties in flushing the machining area. The phenomena take place especially when the hole is deep.

In the EDD process, the amount of heat delivered to the workpiece material and into the material structure affects the removal process. The low thermal conductivity of the Inconel 718 superalloy and its increase with the increase in temperature leads to a decrease in the accuracy of drilling holes. When a lower current amplitude is applied (*I* = 2.66 A) and a similarly appropriate pulse time duration (*t_on_* = 775 µs), a significant amount of re-solidified material occurs on the edge of the hole entrance (Figure 13b). This is the result of the heating of a greater area of material by extending the pulse time of a single pulse, and the energy delivered to the workpiece material is too small to remove material due to the lower current amplitude; however, the application of a longer pulse time, *t_on_* = 550−999 µs, in combination with a higher current amplitude, *I* = 3.99−4.65 A, affects the improvement in the hole accuracy (obtained values of *SG* ≤ 100 µm) (Figure 14b); re-solidified material is absent in this case. Usually, using the longer pulse time worsens the homogeneity of the edge of the entrance to the hole and the accuracy of holes drilled by using the EDD process. In the case of applying the Inconel 718 superalloy as a workpiece material, using longer pulse times and higher current amplitudes is favorable.

In addition, too little energy delivered to the workpiece material and an ineffective flushing of the gap area (among others by applying lower tool electrode rotation *n* = 100 rpm) lead to process instability and lower process efficiency (the drilling speed about 2–3 µm/s). This is the result of short circuits and/or arc discharges during the single pulse time, which are noted in the recorded voltage and current waveforms (Figure 7a,b and Figure 16b).

Using deionized water as a working fluid in combination with a higher current amplitude contributes to the faster heating of deionized water in the gap area, increasing its electrical conductivity. Then, the EDD process is accompanied by electrochemical dissolution, increasing the process efficiency and dimensional accuracy of holes. The reinforced electrochemical reactions during the process are observed by the presence of cavities in the area around the top diameter when applying *I* ≥ 3.99 A and *t_on_* ≥ 550 µs.

The low thermal conductivity of Inconel 718 causes—when the hole is deep and difficulties in effectively flushing the gap area occur—a significant amount of heat to not penetrate the workpiece material. As such, a significant amount of heat affects the machining area and penetrates the tool electrode material. This allows debris to melt and form chains, which are more difficult to remove from the narrow gap area. Secondary discharges can also cause an excessive decrease in the dimensional and shape accuracy of the hole (causing the conicity shape of holes) and the heat penetrating the tool material increases its wear.

The electrical discharge machining process is one of the most effective methods of machining “difficult-to-cut” materials, including the chromo-nickel superalloys, according to the analyzed scientific articles, but the thermophysical properties of these superalloys mean that optimization of the process parameters is still required to obtain a satisfactory process efficiency and hole accuracy.

## 5. Conclusions

In this study, the EDD investigation of the Inconel 718 superalloy was analyzed. Based on the analysis of the results, the following conclusions are determined:

1. The properties of the Inconel 718 superalloy significantly affect its machinability when using the EDD process. Due to the low thermal conductivity of this superalloy, high energy should be delivered to the workpiece material. For that reason, applying a higher current amplitude (*I* ≥ 3.99 A) in combination with an extended pulse time (*t_on_* ≥ 550 µs) provides a satisfactory hole accuracy (*SG* ≤ 100 µm), homogeneity of the hole entrance edge without re-solidified material, and a depth-to-diameter ratio of about 19.
2. For low values of the process parameters, such as current amplitude (*I* = 2.00–2.66 A), the open voltage (*U* = 60 V), and the tool electrode rotation (*n* = 100 rpm), during the single pulse time, short circuits and/or arc discharges occur. This causes a decrease in process efficiency (values of the drilling speed are about 2–3 µm/s) and a decrease in the hole dimensional accuracy (values of the side gap thickness are about 200 µm or greater).
3. Further experimental research is required to improve the machinability of Inconel 718 with the use of EDM, to appropriately handle the complex nature of the phenomena within the gap area during the EDM process (especially in the case of drilling deep holes with a diameter of less than 1 mm), while also dealing with its thermophysical properties.

We have seen that the thermophysical properties of Inconel 718 contribute to the difficulties in its machinability with the use of EDM to reach a satisfactory process performance and high hole accuracy. This indicates that the electrical discharge drilling of micro-holes in this superalloy also constitutes a challenge (especially when selecting appropriate values for machining parameters). In the case where nickel-based superalloys are used as a working material, we suggest further research into the influence of more input parameters on the process performance. Further experimental research with this approach should be carried out to improve the machinability of the Inconel 718 superalloy using this method, particularly in cases where conventional methods are unreliable.

## Figures and Tables

**Table 1:** The chemical composition of Inconel 718 (wt. %).

Ni	Cr	Fe	Nb	Mo	Ti	Al	Co	Mn	C	Si	P
50.0–55.0	17.0–21.0	Balance	4.75–5.5	2.8–3.3	0.65–1.15	0.2–0.8	<1.0	<0.35	<0.08	<0.35	<0.015

**Table 2:** Thermophysical properties for copper [36].

Temperature (°K)	Density (kg/m^3^)	Thermal Conductivity (W/(m∙°K))	Heat Capacity (J/(kg∙°K))
293.15	8930	395.5	381
873.15	8700	344.2	456

**Table 3:** Machining parameters.

Input Parameters	Output Parameters
Open voltage, *U* (V)	Drilling speed *v* (μm/s)
Pulse time, *t_on_* (μs)	Linear tool wear *LTW* (%)
Current amplitude, *I* (A)	Side gap thickness, *SG* (µm)
Inlet dielectric fluid pressure, *p* (MPa)	Aspect ratio hole, *AR*
Tube electrode rotation, *n* (rpm)	

**Table 4:** Process parameters and their levels.

EDD Parameters	Level					
	**1**	**2**		**3**		**4**
*U* (V)	60	80		100		120
	**1**	**2**	**3**		**4**	**5**
*t_on_* (µs)	100	325	550		775	999
*I* (A)	2.00	2.66	3.33		3.99	4.65
*p* (MPa)	5	6	7		8	9
*n* (rpm)	100	200	300		400	500

**Table 5:** Research plan and the results of the experiments. * the blind hole.

Exp. No.	EDD Parameters					Measured Values				
	*U* (V)	*t_on_* (µs)	*I* (A)	*p* (MPa)	*n* (rpm)	*D_top_* (μm)	*D_bottom_* (μm)	*h (*μm)	*h_tool_* (µm)	*t_drilling_ (min)*
1 *	80	325	2.66	6	400	705	400	8783	1370	2700
2 *	80	325	2.66	8	200	656	400	8680	1717	2700
3 *	80	325	3.99	6	200	629	400	9704	2223	2700
4	80	325	3.99	8	400	752	429	10,000	1519	1484
5 *	80	775	2.66	6	200	582	400	7586	1838	2700
6 *	80	775	2.66	8	400	711	400	9504	3070	2700
7	80	775	3.99	6	400	527	441	10,000	2426	1075
8	80	775	3.99	8	200	559	473	10,000	2315	1309
9	100	325	2.66	6	200	522	478	10,000	1630	2700
10	100	325	2.66	8	400	743	422	10,000	1448	1791
11	100	325	3.99	6	400	604	456	10,000	2116	1075
12	100	325	3.99	8	200	587	471	10,000	2309	1908
13	100	775	2.66	6	400	835	528	10,000	3124	1792
14	100	775	2.66	8	200	668	465	10,000	2880	2365
15	100	775	3.99	6	200	612	434	10,000	2860	1600
16	100	775	3.99	8	400	536	495	10,000	2781	812
17 *	60	550	3.33	7	300	835	400	7258	2944	2700
18	120	550	3.33	7	300	668	544	10,000	2638	1429
19	100	100	3.33	7	300	727	408	10,000	2496	2700
20	100	999	3.33	7	300	645	475	10,000	3582	1971
21 *	100	550	2.00	7	300	734	400	8818	3197	2700
22	100	550	4.65	7	300	567	470	10,000	2787	825
23	100	550	3.33	5	300	707	467	10,000	3207	1687
24	100	550	3.33	9	300	657	464	10,000	2826	1302
25 *	100	550	3.33	7	100	1015	400	6179	1896	2700
26	100	550	3.33	7	500	638	503	10,000	2477	1247
27	100	550	3.33	7	300	797	486	10,000	2645	1273
28	100	550	3.33	7	300	857	520	10,000	2833	2342
29	100	550	3.33	7	300	723	452	10,000	2973	1875
30	100	550	3.33	7	300	678	461	10,000	2946	1699
31	100	550	3.33	7	300	673	468	10,000	2705	1971
32	100	550	3.33	7	300	656	432	10,000	2850	1906

**Table 6:** Machining conditions for the electrochemical machining process (ECM).

Machining Parameter	Value/Characteristic
Open voltage, *U* (V)	25
Pulse time, *t_on_* (µs)	300
Feed rate, *v_f_* (µm/min)	25
Test time, *t_m_* (min)	10
Initial interelectrode gap size, *S*_0_ (µm)	50
Inlet working fluid pressure, *p* (MPa)	5
Workpiece material	Inconel 718
Working fluid	Deionized water with electrical conductivity 5 µS/cm
Tool electrode	Single-channel, outer diameter 1.00 mm

## Nomenclature

EDM/EDD	Electrical discharge machining/drilling
ECM	Electrochemical machining
SEM	Scanning electron microscope
*U*	Open voltage, (V)
*t*_*on*_	Pulse time, (µs)
*I*	Current amplitude, (A)
*p*	Inlet dielectric fluid pressure, (MPa)
*n*	Tube electrode rotation, (rpm)
*S*_0_	Initial interelectrode gap thickness, (µm)
*t*_*drilling*_	Drilling time pf each hole, (min)
*t*_*off*_	Pulse off time, (µs)
*h*	The hole depth, (µm)
*h*_*tool*_	The shortening of the electrode, (µm)
*D*_*average*_	The average of the hole diameter, (µm)
*D*_*top*_	The average top diameter, (µm)
*D*_*bottom*_	The average bottom diameter, (µm)
*D*_*tool*_	Outer diameter of the tool electrode, (µm)
*v*_*f*_	Feed rate, (µm/min)
*t*_*m*_	The ECM test time, (min)
*std*	The standard deviation
*v*	Drilling speed, (µm/s)
*LTW*	Linear tool wear, (%)
*SG*	Side gap thickness, (µm)
*AR*	Aspect ratio hole
